# Molecular Identification and Appraisal of the Genetic Variation of *Taenia saginata* in Central Regions of Vietnam

**DOI:** 10.3390/life12010070

**Published:** 2022-01-04

**Authors:** Giang Tran Thi, Ilenia Azzena, Fabio Scarpa, Piero Cossu, Cong Danh Le, Phuong Anh Ton Nu, Thi Minh Chau Ngo, Daria Sanna, Marco Casu

**Affiliations:** 1 Department of Parasitology, Hue University of Medicine and Pharmacy, Hue University, 06 Ngo Quyen Street, Hue City 4200, Vietnam; ttgiang.med@hueuni.edu.vn (G.T.T.); tnphuonganh@hueuni.edu.vn (P.A.T.N.); ntmchau@hueuni.edu.vn (T.M.C.N.); 2 Dipartimento di Medicina Veterinaria, Università di Sassari, Via Vienna 2, 07100 Sassari, Italy; iazzena@uniss.it (I.A.); fscarpa@uniss.it (F.S.); picossu@uniss.it (P.C.); marcasu@uniss.it (M.C.); 3 Dipartimento di Scienze Biomediche, Università di Sassari, Viale San Pietro 43/B, 07100 Sassari, Italy; 4 Traditional Medicine Hospital of Thua Thien Hue Province, Hue City 4200, Vietnam; congdanhbvyhcthue@gmail.com

**Keywords:** cestodes, *Taenia* spp., beef tapeworm, Cytochrome c Oxidase subunit I, Asia

## Abstract

*Taenia saginata* is a globally distributed tapeworm responsible for human taeniasis due to the ingestion of raw or undercooked beef. *T. saginata* is present in several Asian countries, including China, Thailand, Lao PDR, Cambodia, and Vietnam, but little is known about its genetic variation. Studying the tapeworm’s phylogeographic patterns is crucial to better understanding their association with the geographic distribution of taeniasis/cysticercosis in human populations. In the present study, 38 specimens of this putative species were collected in central regions of Vietnam and analysed using the mitochondrial gene Cytochrome c Oxidase subunit I (COI) as a molecular marker to assess the correct species identification and investigate the level of genetic variation at different geographic scales. Phylogenetic and phylogeographic analyses were carried out on a dataset that included COI sequences from Vietnamese specimens and from all conspecifics available in GenBank to date. The results showed that the collected Vietnamese specimens belonged to the species *T. saginata*. In Southeast Asia, signs of a possible founder effect were discovered, with the most common haplotypes frequent and present in many countries, except Lao PDR, which shares its most common haplotype only with individuals from Thailand. Remarkably, a unique taxonomic entity was found worldwide, even though the available COI sequences of *T. saginata* belonging to non-Asiatic countries are, at present, limited. Therefore, future studies including more COI sequences from a higher number of countries and the use of a combined molecular approach with multiple genetic markers would be useful to provide deeper insight into the global genetic variation of this species.

## 1. Introduction

Human taeniasis is a parasitic zoonosis caused by infection of helminth agents belonging to the genus *Taenia* (Linneus 1758) (Cyclophyllidea, *Taeniidae*). *Taenia saginata*, *Taenia solium*, and *Taenia asiatica* are responsible for human taeniasis. Among them, *T. saginata* and *T. solium* are distributed globally, while *T. asiatica* is mostly distributed in Asian countries [1,2,3,4,5]. The symptomatology of infection is broad, but the most frequent and distinctive symptoms are the discharge of proglottids, abdominal pain, weight loss, nausea, and fever [6]. Interestingly, *T. saginata*, *T. solium*, and *T. asiatica* are unique among tapeworms for their life cycle since humans act as their only definitive host. 

In general, the *Taenia* life cycle is based on predator-prey interactions. Faeces, containing helminth eggs, are released into the environment by definitive hosts (carnivorous or omnivorous predators) and ingested by intermediate hosts (herbivorous prey) which become infected. Specifically, when a predator ingests infected meat, the tapeworm completes its life cycle in the intestinal tract of the host and produces egg-filled proglottids, which are released into the environment inside faeces [5,7], where they can be viable for several weeks or even months [2,8]. The eggs released contain oncospheres (a larval form of *Taenia*) which passively infect intermediate hosts when ingested, migrating from the small intestine to skeletal muscle and developing into cysticerci (an intermediate stage that evolves into adult tapeworms inside the human intestinal tract) [2,3,9].

For *T. saginata*, human infection occurs through the ingestion of raw or undercooked beef infected by cysticerci, while *T. solium* and *T. asiatica* human infections occur as a consequence of ingesting infected pork [2,9,10]. 

Stringent socio-economic conditions and the high level of meat inspections allowed European countries to completely eradicate *T. solium* and partially eradicate *T. saginata*. In contrast, the three species are endemic on the Asian mainland, representing a problem in the economy not only in terms of livestock and food production losses, but also for human health (e.g., cysticercosis and neurocysticercosis) [9]. These species coexist in Korea, Taiwan, China, Lao PDR, Thailand [1,5,11], and Vietnam [12], where human taeniasis cases were reported in more than 50 of the 63 provinces. 

As it is difficult to correctly distinguish between *Taenia* species, based on adult tapeworm morphology (isolated in hospitalized patients), molecular techniques are used to ensure a correct taxonomic identification [11]. This method provides a solution to solve the issue of identifying tapeworms, which is essential for diagnosis, treating and controlling taeniasis/cysticercosis. In the present study, we performed a molecular species identification on tapeworm specimens collected in central Vietnam which were tentatively morphologically attributable to *Taenia* sp. after collection. 

We identified tapeworm specimens based on the Cytochrome c Oxidase subunit I (COI) gene sequence, which is also used as a common marker to infer phylogenetic relationships among cestode species [4,5]. However, other molecular markers, such as 28s, 18s and ITS, could be used to study phylogenetic relationships between cestodes [13,14,15,16].

The present study aimed to: (i) identify the species of cestodes found in Vietnamese patients, (ii) investigate the levels of genetic variation in the specimens isolated in Vietnam, and (iii) investigate, if present, the genetic structure of this species among populations, thus further comparing our data to those from other worldwide and Asian countries using the mitochondrial COI gene as a molecular marker.

## 2. Materials and Methods

### 2.1. Sample Collection

**Figure 1:**
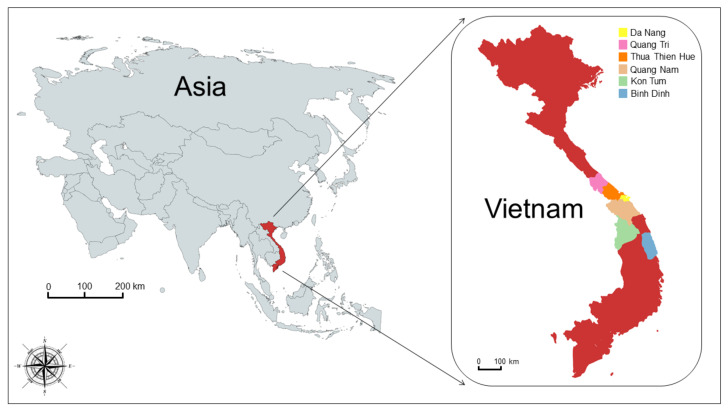
Map of samples’ collection sites. The map shows the geographical origin of Vietnamese sequences which were isolated in the present study.

Thirty-eight adult specimens (gravid proglottids) of *Taenia* spp. were collected from six different regions of central Vietnam between August 2019 and June 2020 (see Table 1 and Figure 1) from hospitalized patients from the Traditional Medicine Hospital of Thua Thien Hue Province (n = 8) and Hue University of Medicine and Pharmacy Hospital (n = 30). Among them, 16 were males, between 5 and 77 years old, and 22 patients were females, between 5 and 71 years old (see Table 2). After treatment, patients were monitored for a period of 3–4 months before being declared completely cured. 

The 38 specimens obtained from patients were firstly identified as *Taenia* sp. on the basis of their morphologic traits: white and flat, approximately 1.5–2 cm long and 0.5 cm wide. Proglottids were not stained to estimate the number of uterine branches as it is difficult to perform a correct species attribution using this method, especially when it is necessary to discriminate between *T. saginata* (14–32 branches) and *T. asiatica* (12–26 branches). 

### 2.2. Diagnostic Molecular Analysis

Total genomic DNA was isolated from a portion of muscle tissue using the Qiagen DNeasy Blood & Tissue Kits (Qiagen, Hilden, Germany) following the supplier’s instructions. After extraction, DNA was stored as a solution at 4 °C. Sample quality and DNA concentration were quantified using the NanoDrop™ 2000 Spectrophotometers (by Thermo Scientific; Waltham, MA, USA), which showed an average yield of approximately 248 ng/µL. A fragment of subunit I of the mitochondrial Cytochrome c Oxidase gene (COI) was amplified by standard PCR using primers, cox1 (forward) (5′-CATGGAATAATAATGATTTTC-3′) and cox1 (reverse) (5′-ACAGTACACACAATTTTAAC-3′) [13]. All PCRs were carried out in a total volume of 25 µL. On average, 10 ng of total genomic DNA were combined with 0.6 µM of each primer and one pellet of PuReTaq Ready-To-Go PCR beads (GE Healthcare, Wauwatosa, WI, USA) containing stabilizers, bovine serum albumin (BSA), deoxynucleotide triphosphates (dNTPs), 2.5 units of PuReTaq DNA polymerase, and reaction buffer.

When a bead was reconstituted to a 25 µL final volume, the concentration of each dNTP and MgCl_2_ was set at 200 µM and 1.5 mM, respectively. PCRs were performed in a GeneAmp PCR System 9700 Thermal Cycler (Applied Biosystems, Waltham, MA, USA), programmed as follows: 1 cycle of 4 min at 94 °C, 35 cycles of 30 s at 94 °C, 30 s 56 °C and, 30 s at 72 °C. At the end, a post-treatment of 10 min at 72 °C and a final cooling at 4 °C were carried out. Both positive and negative controls were used to test the effectiveness of the PCR protocols, and the absence of possible contaminations. Electrophoresis was carried out on 2% agarose gels, prepared using 1x TAE buffer (Tris-Acetate-EDTA, pH 8.3) and stained with Gel Red Nucleic Acid Stain (Biotium Inc., Fremont, CA, USA). PCR products were purified by ExoSAP-IT (USB Corporation, Cleveland, OH, USA) and sequenced for forward and reverse strands (by means of the same primers used for PCR), using an external sequencing core service (Macrogen Europe, Amsterdam, The Netherlands).

### 2.3. Phylogeographic and Phylogenetic Analysis

Thirty-eight newly-generated sequences of COI fragments were aligned using the package Clustal Omega [17], available at https://www.ebi.ac.uk/Tools/msa/clustalo/ (last access: 10 October 2021) and deposited in GenBank (see Table 1 for GenBank accession numbers). To perform molecular analyses to consider our data in a wider geographic context, we constructed two datasets: one including all the species of *Taenia* globally distributed, available on GenBank to date, and a second one including only the sequences of *T. saginata* from Asian countries, available on GenBank to date (last access: 24 August 2021) (See Table S1 for GenBank accession number). Both of these two datasets include a sequence of *Echinococcus granulosus* (MN787534) as an outgroup.

Levels of genetic variation among sequences were assessed estimating the number of polymorphic sites (*S*), number of haplotypes (*H*), nucleotide diversity (*π*), and haplotype diversity (*hd*), using the software package DnaSP 6.12.03 [18].

Two median-joining networks [19] were constructed using the software package Network 10.2.0.0 (www.fluxus-engineering.com) (Colchester, UK) to infer the genetic relationships among Vietnamese and Asian haplotypes. The transitions and transversions were equally weighed. Due to the lack of knowledge regarding the possible occurrence of retromutation events, the same weight (10) was assigned to all the observed polymorphisms. 

Phylogenetic relationships among specimens were investigated using Bayesian inference (BI) by means of the software MrBayes 3.2.7 [20].

The simplest evolutionary model that best fits the sequence data was detected using the software JmodelTest 2.1.7 [21] and PartitionFinder 2.1.1 [22]. These softwares provided the same result and, in accordance with the best-fitting model, the runs in MrBayes were performed by setting the following model parameters: NST = 6, rates = invgamma, ngammacat = 4. Two independent runs, each consisting of four Metropolis-Coupled MCMC chains (one cold and three heated chains), were run simultaneously for 5 million generations, sampling trees every 1000 generations. 

In order to test the convergence of chains, we checked that the average standard deviation of split frequencies (ASDSF) approached 0 [20], and the potential scale reduction factor (PSRF) was around 1 [23], following Scarpa et al. [24].

The phylogenetic tree was visualized and edited using FigTree 1.4.0 (available at http://tree.bio.ed.ac.uk/software/figtree/) (last access: 10 October 2021) (Edinburgh, UK).

In order to verify the taxonomic assessment of each sequence in the dataset, three different methods of species delimitation were used. The use of different methods, based on different criteria and algorithms, is crucial for a conservative approach, which avoids attribution of sequences to erroneous taxonomic entities. In the present study, only results corroborated by all the used species delimitation methods were considered as consistent and well-supported. The first method used is the PTP (Poisson Tree Processes) model and its Bayesian implementation bPTP [25]. PTP/bPTP work on the phylogenetic species concept (PSC) using the number of substitutions in tree branches to assess the speciation rate. The method tests for a significant shift in the substitution rate, which is indicative of the switch from between-species to within-species processes. Species delimitation was performed by means of the bPTP web server (available at http://species.h-its.org/ptp/) (last access: 10 October 2021) (Heidelberg and Karlsruhe, Germany) by using the Bayesian phylogenetic species tree as input file, with default options and 500,000 MCMC generations. Chain convergence was verified by visualizing the likelihood plot. If convergence occurred, the chain should stay at high likelihood locations most of the time during the run.

The second method was the Nucleotide Divergence Threshold (NDT), which was implemented by means of a customized script written in the R statistical environment proposed by Scarpa et al. [26]. The script ranks specimens into taxonomic entities applying the fixed threshold of 2% given by Hebert et al. [27] for DNA barcodes, using a pairwise Kimura two-parameter model (K2P) genetic distances matrix [28]. In the present study the K2P was chosen as it is recommended to estimate genetic distances that will be used for taxonomic purposes. 

The last used method was the Assemble Species by Automatic Partitioning (ASAP) [29] which was performed by using the *p*-distance model (as substitution model to calculate the distances matrix), selecting default options. The ASAP is a fully exploratory method, and it does not require any kind of a priori knowledge. The species hypothesis was accepted by Puillandre et al. [29], within the list of the best partitions valuating their gap-width score, *p*-value and threshold distance. 

To identify potential subgroups within the genetic clusters and to determine the dissimilarity represented by the genetic variation among sequences, a Principal Coordinates Analysis (PCoA) was performed using GenAlEX 6.5 [30] on a matrix of pairwise genetic distances corrected according to the Kimura two-parameter model (K2P) [28]. 

Furthermore, in order to verify the occurrence of genetic association between genetic variability and host age, the PCoA was also performed grouping patients according to their age, thus using three age classes (0–30; 30–50; ≥50). 

## 3. Results

Thirty-eight sequences of the initial portion of the COI gene were obtained from Vietnamese specimens (Table 1). This dataset showed 18 polymorphic sites which defined 15 haplotypes (see Table 3 for details on genetic divergence estimates). 

All the sequences obtained from the Vietnamese specimens showed a 100% of genetic identity match with the species *T. saginata* with the Basic Local Alignment Search Tool (BLAST) analysis implemented in the GenBank nucleotide database (www.ncbi.nlm.nih.gov, accessed on 21 July 2020).

**Figure A1:**
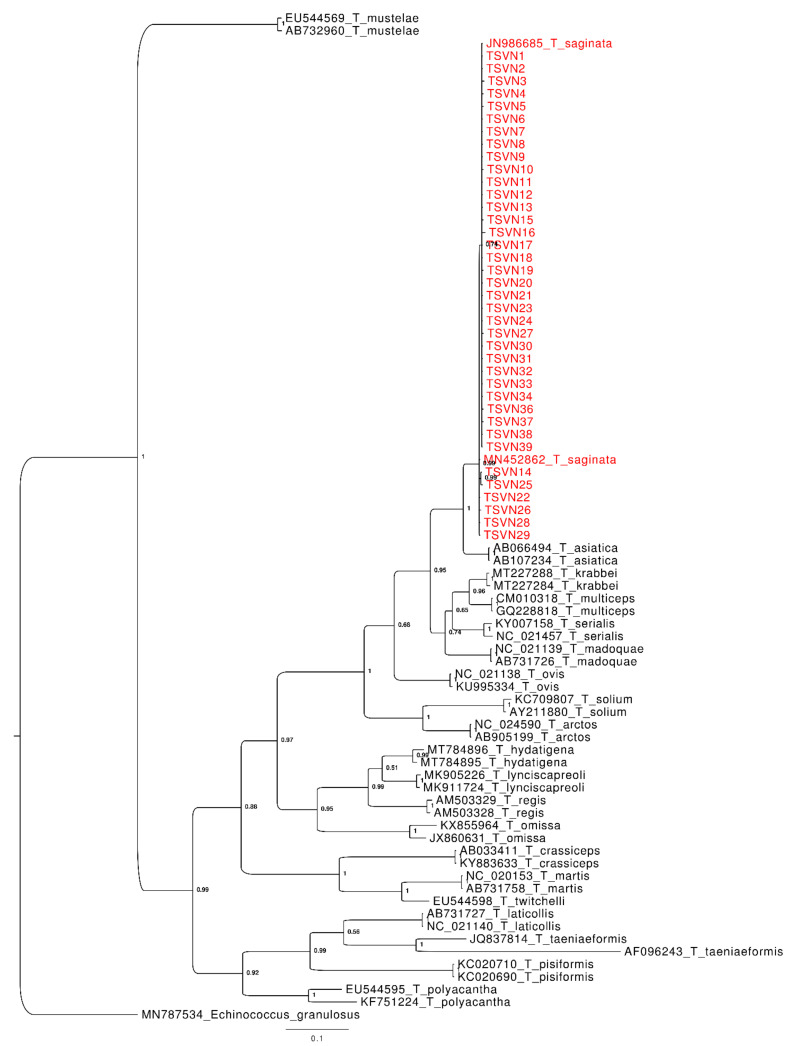
Bayesian phylogenetic tree based on the *Taenia* spp. COI sequences dataset. Posterior probability values are reported at each node. Vietnamese sample codes are as reported in the Table 1 of the main text.

To correctly assess the species of samples collected during the present study, before proceeding with phylogeographic inferences, two analyses were performed on a dataset that included the COI sequences of all the species belonging to the genus *Taenia* available in the GenBank database to date. A total of 80 sequences, representative of 21 *Taenia* species (at least two individuals per species when possible) and one outgroup (*Echinococcus granulosus*), were used (see in Appendix A, Table A1 and Figure A1 for details on species and GenBank accession numbers).

First a phylogenetic tree analysis (see in Appendix A, Figure A1) was performed, which showed a unique, well-supported monophyletic cluster, that included the sequences obtained in the present study, along with those of *T. saginata* from GenBank. This genetic clade is characterized by an extended polytomy with some internal well-supported sub-clusters including only a few sequences, and it represents the sister taxon of *T. asiatica*. No relevant structuring based on the geographic distribution of hosts was found among the sequences.

For the second analysis performed on the *Taenia* species dataset, three species delimitation methods (see in Appendix A, Table A1 for details) were used. In general, all the methods were consistent, evidencing a number of taxonomic entities corresponding to the number of species present in the dataset. The only exceptions were represented by *T. hydatigena*, *T. omissa*, *T. polyacantha, T. serialis* and *T. taeniaeformis,* whose sequences split into different taxonomic entities by some of the methods (see in Appendix A, Table A1 for details) as a possible consequence of the high discrimination capacity of these methods. In accordance with BLAST and phylogenetic tree analyses, all methods of species delimitation suggest that the sequences isolated from Vietnamese samples in the present study belong to the taxonomic entity of *T. saginata*.

After reaching the correct taxonomic identification for Vietnamese cestodes, further analyses were performed to infer the levels of genetic variation among populations in Vietnam and other countries.

**Figure 2:**
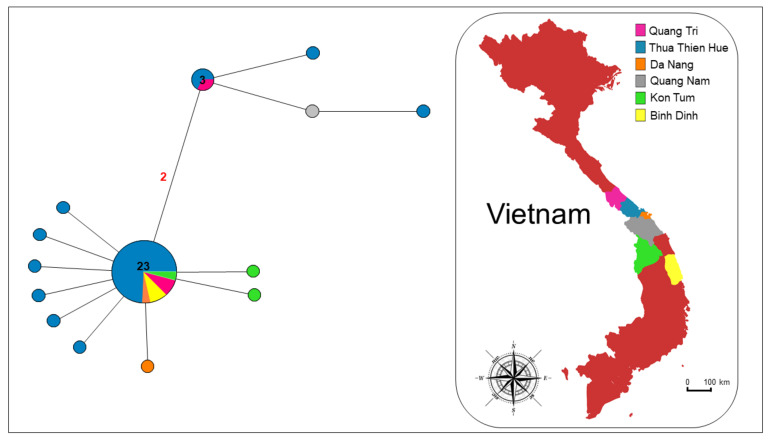
Median-joining network analysis. The network includes COI Vietnamese sequences from the present study. The number of mutations between sequences that are greater than n = 1 are reported on network branches. Additionally, the number of individuals showing the same haplotype that is greater than n = 1 is reported inside the spot.

The network analysis performed on the Vietnamese samples (Figure 2) showed the presence of two clusters. One cluster is more common and characterized by a well-defined star-like shape with a central highly diffused haplotype including about 60.5% of the sequences analysed. 

Thirteen haplotypes diverged from the most common haplotype, by accumulation of one-to-four-point mutations. The second cluster is less widespread, including 15.8 % (six individuals) of the sequences analysed and shows an almost star-like shape, with one common haplotype and three derivatives. Specimens coming from the Thua Thien Hue (4), Quang Nam (1) and Quang Tri (1) provinces belong to this cluster.

The same Vietnamese dataset was used to evaluate whether genetic structuring was present among samples based on the age of the hosts (patient age are provided in Table 2) by performing a PCoA (see in the supplementary material, Figure S1 and Table S2). Samples were considered depending on the age (in years) of patients, three age groups were used (0–30, 30–50, ≥50). Results evidenced the absence of genetic structuring among sequences, also evidenced by the low percentage of variation explained by the first two axes (PCoA 1: 41.99%; PCoA 2: 10.97%), thus suggesting a lack of association between genetic variation and the age of infected patients. 

**Figure 3:**
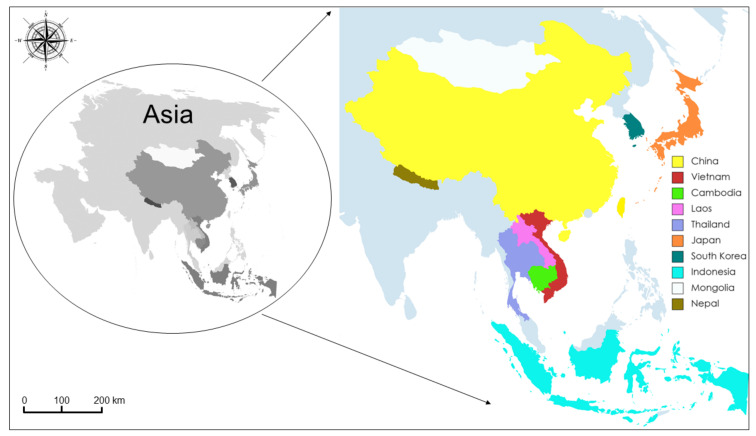
Map of the Asian sequences’ distribution. The map shows the geographical sites where the Asian sequences from GenBank (the accession numbers are reported in the supplementary Table S1), which were used in the present study, were collected.

Another dataset, including the Vietnamese COI sequences obtained in the present study, along with those belonging to *T. saginata* from other Asian isolates deposited in GenBank, was constructed to perform a second network analysis (see Figure 3 and the supplementary Table S1 for details). 

This dataset includes 182 sequences and comprises 51 polymorphic sites, which define 50 haplotypes. In contrast, a very low nucleotide diversity (*π*) was found (see Table 3 for details on genetic divergence estimates).

**Figure 4:**
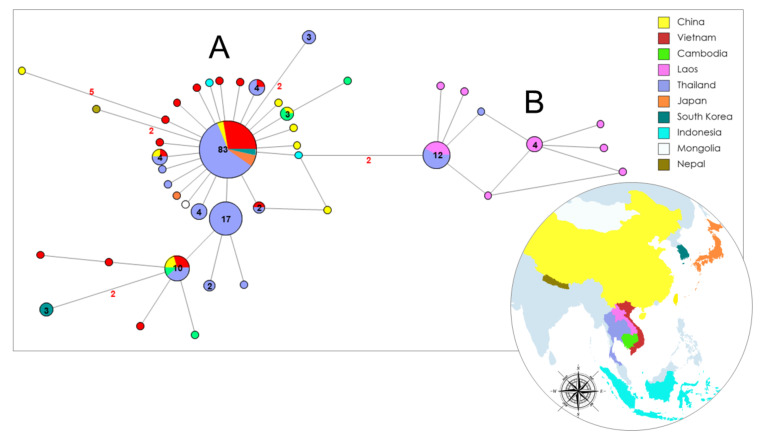
Median-joining network analysis. The network includes COI Vietnamese sequences from the present study along with those belonging to *Taenia saginata* from other Asian isolates which are available in GenBank (the accession numbers are reported in the supplementary Table S1). The number of mutations between sequences that are greater than n = 1 are reported on network branches. Additionally, the number of individuals showing the same haplotype that is greater than n = 1 is reported inside the spot.

The analyses revealed the presence of two clusters (Figure 4): cluster A, which was more common and spread in all the Asian countries analysed, except Lao PDR, and cluster B, which was only present in Thailand and Lao PDR. Cluster A is characterized by a well-defined star-like shape with a common haplotype shared by 45.6% of the total sequences. Thai samples show the principal and derivative haplotypes of both clusters, and Lao PDR samples share only one haplotype with eight sequences from Thailand. New derivative haplotypes in the network diverged from the most common ones by the accumulation of one point mutation. Ten of them had never been previously reported and were isolated in four of the six Vietnamese provinces which were analysed in the present study (six from Thua Thien Hue, two from Kon Tum, one from Da Nang, and one from Quang Nam). Two not-resolved reticulations are present in the network, but negligible, as the relationships between the haplotypes are very clear.

A third dataset was also constructed upon which to perform phylogenetic analyses and evaluate the data obtained in the present study in a wider geographic context. It included the Vietnamese sequences obtained in the present study and those corresponding to the same portion of the COI gene of *T. saginata* isolated worldwide and deposited in GenBank (see Table S1 for accession numbers). 

This dataset included 202, 1013 bp-long, sequences of *T. saginata* from 18 countries (see supplementary Table S1 for details). Among them, 60 polymorphic sites were retrieved, corresponding to 57 haplotypes. Moderate high levels of polymorphic sites (*S*) and haplotype diversity (*hd*) were found, while a very low values of nucleotide diversity (*π*) was found (see Table 3 for further details on genetic divergence estimates).

Accordingly, the PTP/bPTP, NDT, and ASAP species delimitation methods were consistent in grouping all *T. saginata* COI sequences in a unique worldwide distributed taxonomic entity. Indeed, only one taxonomic entity for all the sequences was evidenced by all the methods of species delimitation used (see in Appendix A, Table A1). 

**Figure 5:**
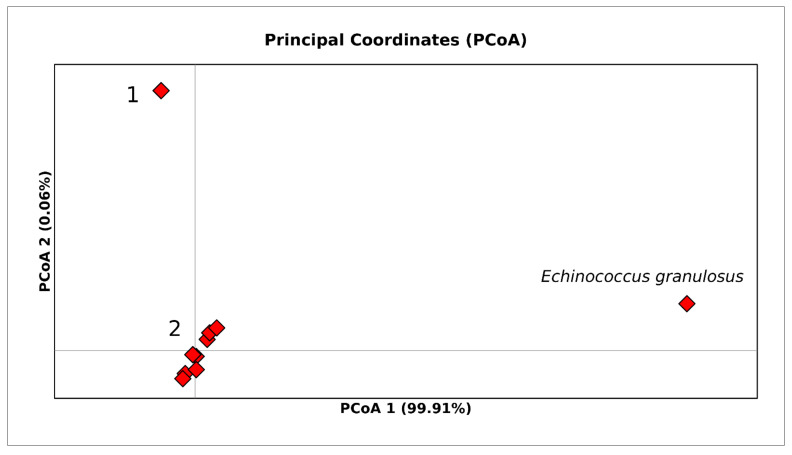
PCoA performed on the whole world *Taenia saginata* COI dataset. Bi-dimensional plot shows the genetic differentiation among specimens due to the nucleotide substitutions per site found in the dataset. The number 1 inside the figure stands for Group 1 and the number 2 stands for Group 2 (see the supplementary Table S3 for details). PCoA1 (axis X) accounts for 99.91% of variation, while PCoA2 (axis Y) accounts for 0.06% of variation.

To further verify whether the few internal well-supported clusters evidenced in the phylogenetic tree (see Figure A1 in Appendix A) were consistent with the possible occurrence of different taxonomic entities within *T. saginata*, a PCoA (Figure 5) was performed, including in the analysed dataset the outgroup that was also used for the phylogenetic tree. 

PCoA results showed the occurrence of two closely related genetic groups (1 and 2 in Figure 5) along the axis X (PCoA1), for *T. saginata* which were equally divergent from the outgroup *E. granulosus*, thus corroborating the general homogeneity evidenced within *T. saginata* by previous analyses. In particular, the first axis (PCoA 1) accounted for 99.91% of the variation, while the second axis (PCoA 2) accounted for 0.06% of the variation. Group 1 was distributed worldwide and included 98.5% of the sequences, while Group 2 included only 1.5% of the total sequences (three strains isolated in Thailand in 2010 and 2016; see the supplementary Table S3 for details). 

## 4. Discussion

This study found that the 38 *Taenia* specimens collected in central Vietnam belong to the species *T. saginata*. Inferences on the genetic variation of this species at different geographic scales—within Vietnam, through Asia, and worldwide—are also provided, using the mitochondrial COI sequences currently available in GenBank. 

Correct taxonomic attribution of *Taenia* spp. specimens, based only on morphological characteristics, is very difficult to be performed, especially when it is necessary to differentiate between *T. saginata* and *T. asiatica*. Indeed, the main morphological feature taken into account to distinguish between *Taenia* species is the proglottids, and the aforementioned species are closely related due to the similarity of their proglottids [31,32,33,34,35]. Therefore, the use of the mitochondrial COI gene in this study allowed an accurate taxonomic identification of *Taenia* sp. specimens to be conducted. In addition, the use of several molecular species delimitation methods based on different criteria and algorithms allowed us to perform a conservative and robust approach. All the methods used agreed in evidencing that the sequences obtained from Vietnamese samples in the present study belonged to the same taxonomic entity: *Taenia saginata*. 

Extant knowledge about genetic variation in Vietnamese *T. saginata* is limited, and few studies based on mitochondrial markers [2,13] are available. However, analyses of genetic variation based on sequences of *T. saginata* COI gene in Asia were performed for populations from Thailand and Lao PDR [2,13], where a high level of variation was found. This suggests that *T. saginata* might have spread through different routes in Southeast Asia [2]. In accordance with these data, the present study’s results suggest the occurrence of a possible founder effect (as evidenced by star-like shapes in the Network analyses) in Asian countries. Thailand in particular seems to be a hotspot of biodiversity from which *T. saginata* expands into neighboring areas, according to the typical trend of expansion reported for the populations recently originated from a few founders. In fact, either the most common and several derivatives mitochondrial haplotypes were found among the Thai samples, suggesting that genetic variation of tapeworms in Thailand may be representative of one of the first Asian areas colonized by *T. saginata*. Accordingly, the most common COI haplotype found in Thailand and other Asian countries may correspond to one of the oldest mitochondrial variants and it is likely representative of the first lineages introduced to the region. 

Even considering a possible bias due to the high number of Thai samples present in the dataset, this occurrence could be explained in the context of the introduction of *T. saginata* to Asia, where the Northeast area of Thailand and Lao PDR may represent the first centres of infection, according to Sanpool et al. [2]. 

This finding could be explained by the human mediated movements of cattle. Thailand was known as a source of cattle export [36] where transhumance of animals started, passed across the Southern provinces of Laos, and arrived in Vietnam [36,37]. However, it is interesting to note that Lao PDR *Taenia* populations do not seem to have expanded beyond the borders of the country; this may be partly due to the economic conditions of the area, with its predominantly rural-based agriculture and limited cattle export [10]. The few Lao PDR sequences shared with Thailand belong to specimens collected in the Northwest, near the border between Lao PDR and Thailand. In addition, no haplotype is shared between the Vietnamese and Lao PDR *T. saginata* populations. These results could be a consequence of: (i) few collection sites and sequences available from Lao PDR on GenBank; (ii) excessively rapid animal transit during transhumance in Lao PDR; and (iii) genetic drift, leading to the spread of haplotypes in Lao PDR that are uncommon in Thailand. 

The presence in Vietnam of several haplotypes shared with other populations from Asia, may be a consequence of the economic importance of commercial routes between Vietnam and other Southeast Asian countries. Indeed, most of the haplotypes shared between Thailand and Vietnam could be closely related to the commercial movements of cattle for grazing or slaughtering [38]. For a long time, informal cross-border trade between Thailand and Vietnam was difficult to be controlled, especially in the border of transit shared between Lao PDR and the Vietnamese province of Quang Tri [38]. Furthermore, a constant flow of human workers from Vietnam to Thailand has lasted for many centuries. There is no official estimate for the number of Vietnamese migrants in Thailand, as most of them are illegal immigrants, but unofficial estimates suggest the presence of approximately 50,000 Vietnamese workers between 2012 and 2014 [39]. In this context, it is very likely that workers became infected during their tenure in Thailand, but because they were illegally present in Thai territory, they could only be cured after returning to their hometowns in Vietnam. On the other hand, although labor migration, legal or not, also occurs between Vietnam and Laos PDR [37], the results obtained in the present study do not show any haplotype shared between these two countries. In the future, molecular studies with more sequences from Lao PDR could be useful to confirm or refute this trend and to better understand the dynamics of the *T. saginata* genetic flow between Lao PDR and Vietnam. In general, human activities could explain the homogeneity of haplotypes found in Southeast Asia in the present study.

A bias in the frequencies of sequences from outside Asia is present in the worldwide dataset analysed here, which is composed of only 11.58% (n = 19) of non-Asian country sequences. This low number may have influenced the results obtained for the whole world population in the present study, which evidenced a unique monophyletic taxonomic entity for *T. saginata* globally. However, even if this finding is not conclusive for worldwide populations, it could be representative of the real taxonomic status of *T. saginata* in Asia, whose common ancestor seems to have differentiated in 2001.

Interestingly, no trace of the occurrence of *T. solium* was found among the Vietnamese specimens analysed here. This pathogen is responsible for neurocysticercosis which is an infection belonging to the group of Neglected Tropical Diseases (NTDs). This result is in accordance with a previous study by Ng-Nguyen et al. [40] which showed very low *T. solium* infection rates in central Vietnam. Although these findings should be further corroborated by a deeper sampling campaign, they are nonetheless suggestive of a low risk for cysticercosis/neurocysticercosis in the human communities of central Vietnam and also provide evidence that the introduction of proper prevention and management strategies in these Vietnamese areas may have yielded optimal results in controlling the spread of tapeworm infection. 

In conclusion, the present study sheds further light on the origin and spread of *T. saginata* in Asia as well as in central Vietnam. In the future, further molecular surveys with a wider sampling plan (including sequences from Vietnam and neighboring countries), and the combined use of mitochondrial and nuclear markers will be needed to corroborate the genetic trends evidenced here.

## Figures and Tables

**Table 1:** Sampling plan. The table reports data on the sampling collection and the GenBank accession numbers of the sequences obtained in the present study.

Sample ID	Sampling Site	Species	Host	Genbank Accession #	Sampling Date
TSVN01	Vietnam: Thua Thien Hue	*Taenia saginata*	*Homo sapiens*	OL459841	August 2019
TSVN02	Vietnam: Thua Thien Hue	*Taenia saginata*	*Homo sapiens*	OL459842	August 2019
TSVN04	Vietnam: Thua Thien Hue	*Taenia saginata*	*Homo sapiens*	OL459844	September 2019
TSVN05	Vietnam: Thua Thien Hue	*Taenia saginata*	*Homo sapiens*	OL459845	September 2019
TSVN06	Vietnam: Thua Thien Hue	*Taenia saginata*	*Homo sapiens*	OL459846	September 2019
TSVN07	Vietnam: Thua Thien Hue	*Taenia saginata*	*Homo sapiens*	OL459847	September 2019
TSVN08	Vietnam: Thua Thien Hue	*Taenia saginata*	*Homo sapiens*	OL459848	September 2019
TSVN09	Vietnam: Thua Thien Hue	*Taenia saginata*	*Homo sapiens*	OL459849	September 2019
TSVN10	Vietnam: Thua Thien Hue	*Taenia saginata*	*Homo sapiens*	OL459850	November 2019
TSVN11	Vietnam: Thua Thien Hue	*Taenia saginata*	*Homo sapiens*	OL459851	November 2019
TSVN12	Vietnam: Thua Thien Hue	*Taenia saginata*	*Homo sapiens*	OL459852	November 2019
TSVN15	Vietnam: Thua Thien Hue	*Taenia saginata*	*Homo sapiens*	OL459855	November 2019
TSVN24	Vietnam: Thua Thien Hue	*Taenia saginata*	*Homo sapiens*	OL459864	January 2020
TSVN25	Vietnam: Thua Thien Hue	*Taenia saginata*	*Homo sapiens*	OL459865	January 2020
TSVN26	Vietnam: Thua Thien Hue	*Taenia saginata*	*Homo sapiens*	OL459866	February 2020
TSVN27	Vietnam: Thua Thien Hue	*Taenia saginata*	*Homo sapiens*	OL459867	February 2020
TSVN28	Vietnam: Thua Thien Hue	*Taenia saginata*	*Homo sapiens*	OL459868	February 2020
TSVN29	Vietnam: Thua Thien Hue	*Taenia saginata*	*Homo sapiens*	OL459869	March 2020
TSVN30	Vietnam: Thua Thien Hue	*Taenia saginata*	*Homo sapiens*	OL459870	May 2020
TSVN31	Vietnam: Thua Thien Hue	*Taenia saginata*	*Homo sapiens*	OL459871	May 2020
TSVN32	Vietnam: Thua Thien Hue	*Taenia saginata*	*Homo sapiens*	OL459872	May 2020
TSVN33	Vietnam: Thua Thien Hue	*Taenia saginata*	*Homo sapiens*	OL459873	May 2020
TSVN34	Vietnam: Thua Thien Hue	*Taenia saginata*	*Homo sapiens*	OL459874	May 2020
TSVN36	Vietnam: Thua Thien Hue	*Taenia saginata*	*Homo sapiens*	OL459875	May 2020
TSVN37	Vietnam: Thua Thien Hue	*Taenia saginata*	*Homo sapiens*	OL459876	June 2020
TSVN38	Vietnam: Thua Thien Hue	*Taenia saginata*	*Homo sapiens*	OL459877	June 2020
TSVN39	Vietnam: Thua Thien Hue	*Taenia saginata*	*Homo sapiens*	OL459878	June 2020
TSVN03	Vietnam: Da Nang	*Taenia saginata*	*Homo sapiens*	OL459843	September 2019
TSVN13	Vietnam: Da Nang	*Taenia saginata*	*Homo sapiens*	OL459853	November 2019
TSVN14	Vietnam: Quang Nam	*Taenia saginata*	*Homo sapiens*	OL459854	November 2019
TSVN16	Vietnam: Kon Tum	*Taenia saginata*	*Homo sapiens*	OL459856	December 2019
TSVN19	Vietnam: Kon Tum	*Taenia saginata*	*Homo sapiens*	OL459859	December 2019
TSVN23	Vietnam: Kon Tum	*Taenia saginata*	*Homo sapiens*	OL459863	December 2019
TSVN17	Vietnam: Binh Dinh	*Taenia saginata*	*Homo sapiens*	OL459857	December 2019
TSVN20	Vietnam: Binh Dinh	*Taenia saginata*	*Homo sapiens*	OL459860	December 2019
TSVN18	Vietnam: Quang Tri	*Taenia saginata*	*Homo sapiens*	OL459858	December 2019
TSVN21	Vietnam: Quang Tri	*Taenia saginata*	*Homo sapiens*	OL459861	December 2019
TSVN22	Vietnam: Quang Tri	*Taenia saginata*	*Homo sapiens*	OL459862	December 2019

**Table 2:** Sampling plan. The table reports data of the patients who had taeniasis infections, and were cured between August 2019 and June 2020, from which the *Taenia* sp. specimens used in the present study were collected.

Sample ID	Age	Sex	Sampling Region	Hospital	Collection Year
TSVN01	5	F	Thua Thien Hue	Hue University of Medicine and Pharmacy Hospital	2019
TSVN02	41	M	Thua Thien Hue	Hue University of Medicine and Pharmacy Hospital	2019
TSVN04	71	F	Thua Thien Hue	Hue University of Medicine and Pharmacy Hospital	2019
TSVN05	57	M	Thua Thien Hue	Hue University of Medicine and Pharmacy Hospital	2019
TSVN06	32	M	Thua Thien Hue	Hue University of Medicine and Pharmacy Hospital	2019
TSVN07	48	M	Thua Thien Hue	Hue University of Medicine and Pharmacy Hospital	2019
TSVN08	67	F	Thua Thien Hue	Hue University of Medicine and Pharmacy Hospital	2019
TSVN09	60	F	Thua Thien Hue	Hue University of Medicine and Pharmacy Hospital	2019
TSVN10	27	M	Thua Thien Hue	Hue University of Medicine and Pharmacy Hospital	2019
TSVN11	57	F	Thua Thien Hue	Traditional Medicine Hospital of Thua Thien Hue Province	2019
TSVN12	51	F	Thua Thien Hue	Traditional Medicine Hospital of Thua Thien Hue Province	2019
TSVN15	55	M	Thua Thien Hue	Hue University of Medicine and Pharmacy Hospital	2019
TSVN24	77	M	Thua Thien Hue	Traditional Medicine Hospital of Thua Thien Hue Province	2020
TSVN25	52	F	Thua Thien Hue	Traditional Medicine Hospital of Thua Thien Hue Province	2020
TSVN26	46	M	Thua Thien Hue	Hue University of Medicine and Pharmacy Hospital	2020
TSVN27	66	F	Thua Thien Hue	Hue University of Medicine and Pharmacy Hospital	2020
TSVN28	53	F	Thua Thien Hue	Hue University of Medicine and Pharmacy Hospital	2020
TSVN29	53	F	Thua Thien Hue	Traditional Medicine Hospital of Thua Thien Hue Province	2020
TSVN30	32	M	Thua Thien Hue	Hue University of Medicine and Pharmacy Hospital	2020
TSVN31	24	M	Thua Thien Hue	Hue University of Medicine and Pharmacy Hospital	2020
TSVN32	31	M	Thua Thien Hue	Traditional Medicine Hospital of Thua Thien Hue Province	2020
TSVN33	47	M	Thua Thien Hue	Hue University of Medicine and Pharmacy Hospital	2020
TSVN34	47	M	Thua Thien Hue	Hue University of Medicine and Pharmacy Hospital	2020
TSVN36	56	F	Thua Thien Hue	Traditional Medicine Hospital of Thua Thien Hue Province	2020
TSVN37	27	M	Thua Thien Hue	Traditional Medicine Hospital of Thua Thien Hue Province	2020
TSVN38	36	M	Thua Thien Hue	Hue University of Medicine and Pharmacy Hospital	2020
TSVN39	52	F	Thua Thien Hue	Hue University of Medicine and Pharmacy Hospital	2020
TSVN03	19	F	Da Nang	Hue University of Medicine and Pharmacy Hospital	2019
TSVN13	50	F	Da Nang	Hue University of Medicine and Pharmacy Hospital	2019
TSVN14	38	F	Quang Nam	Hue University of Medicine and Pharmacy Hospital	2019
TSVN16	47	F	Kon Tum	Hue University of Medicine and Pharmacy Hospital	2019
TSVN19	63	F	Kon Tum	Hue University of Medicine and Pharmacy Hospital	2019
TSVN23	46	F	Kon Tum	Hue University of Medicine and Pharmacy Hospital	2019
TSVN17	48	F	Binh Dinh	Hue University of Medicine and Pharmacy Hospital	2019
TSVN20	51	F	Binh Dinh	Hue University of Medicine and Pharmacy Hospital	2019
TSVN18	69	F	Quang Tri	Hue University of Medicine and Pharmacy Hospital	2019
TSVN21	52	F	Quang Tri	Hue University of Medicine and Pharmacy Hospital	2019
TSVN22	5	M	Quang Tri	Hue University of Medicine and Pharmacy Hospital	2019

**Table 3:** Indices of genetic variation. The table reports the estimates of genetic variation for the mitochondrial COI gene dataset. N: sample sizes; bp: fragment size; *S*: number of polymorphic sites; *H*: number of haplotypes; *hd*: haplotype diversity; *π*: nucleotide diversity.

	N	bp	*H*	*S*	*hd*	*π*
Vietnamese COI dataset	38	1013	15	18	0.667	0.00142
Asian COI dataset	182	1013	50	51	0.789	0.00211
Whole world COI dataset	202	1013	57	60	0.794	0.00209

## Data Availability

Sequences obtained in the present study for the mitochondrial Cytochrome c Oxidase subunit I gene isolated in Vietnamese *Taenia saginata* were deposited in the GenBank database under the accession numbers OL459841-OL459878.

## Supplementary Materials

The following are available online at https://www.mdpi.com/article/10.3390/life12010070/s1, Table S1: Sequences from GenBank. The table reports data on the whole world *Taenia saginata* COI dataset of sequences which were used in the present study and are available on GenBank. Table S2: Sequences used for PCoA. The table reports data on Vietnamese *Taenia saginata* COI sequences (from the present study) which were used for PCoA, they are categorized according to the host age. We set three different age groups: 0–30 years identified by the code 30, 30–50 years identified by the code 50 and, ≥50 years identified by the code 50+. Table S3: Sequences for PCoA. The table reports data on the *Taenia saginata* COI sequences which were used for PCoA in the present study and are available on GenBank.

## Appendix A

**Table A1:** Species delimitation methods results based on the analysis of the COI sequences belonging to different *Taenia* species. Specimens with identical numbers within the same column belong to the same taxonomic unit.

Genbank #	Species	NDT	ASAP	PTP/bPTP
MN787534	*Echinococcus granulosus*	1	1	1
JN986685	*Taenia saginata*	2	2	20
MN452862	*Taenia saginata*	2	2	20
AB066494	*Taenia asiatica*	3	3	21
AB107234	*Taenia asiatica*	3	3	21
KC709807	*Taenia solium*	4	4	8
AY211880	*Taenia solium*	4	4	8
NC_024590	*Taenia arctos*	5	5	9
AB905199	*Taenia arctos*	5	5	9
AB033411	*Taenia crassiceps*	6	6	7
KY883633	*Taenia crassiceps*	6	6	7
MT784896	*Taenia hydatigena*	7	7	24
MT784895	*Taenia hydatigena*	8	7	25
MT227288	*Taenia krabbei*	9	8	22
MT227284	*Taenia krabbei*	9	8	22
AB731727	*Taenia laticollis*	10	9	4
NC_021140	*Taenia laticollis*	10	9	4
MK905226	*Taenia lynciscapreoli*	11	10	17
MK911724	*Taenia lynciscapreoli*	11	10	17
NC_021139	*Taenia madoquae*	12	11	16
AB731726	*Taenia madoquae*	12	11	16
NC_020153	*Taenia martis*	13	12	10
AB731758	*Taenia martis*	13	12	10
CM010318	*Taenia multiceps*	14	13	23
GQ228818	*Taenia multiceps*	14	13	23
EU544569	*Taenia mustalae*	15	14	6
AB732960	*Taenia mustalae*	15	14	6
KX855964	*Taenia omissa*	16	15	18
JX860631	*Taenia omissa*	17	16	19
NC_021138	*Taenia ovis*	18	17	13
KU995334	*Taenia ovis*	18	17	13
KC020710	*Taenia pisiformis*	19	18	5
KC020690	*Taenia pisiformis*	19	18	5
EU544595	*Taenia polyacantha*	20	19	14
KF751224	*Taenia polyacantha*	21	20	15
AM503329	*Taenia regis*	22	21	12
AM503328	*Taenia regis*	22	21	12
KY007158	*Taenia serialis*	23	22	26
NC_021457	*Taenia serialis*	23	22	27
EU544598	*Taenia twitchelli*	24	23	11
JQ837814	*Taenia taeniaeformis*	25	24	2
AF096243	*Taenia taeniaeformis*	26	25	3
OL459841	*Taenia* sp. from the present study (TSVN1)	2	2	20
OL459842	*Taenia* sp. from the present study (TSVN2)	2	2	20
OL459843	*Taenia* sp. from the present study (TSVN3)	2	2	20
OL459844	*Taenia* sp. from the present study (TSVN4)	2	2	20
OL459845	*Taenia* sp. from the present study (TSVN5)	2	2	20
OL459846	*Taenia* sp. from the present study (TSVN6)	2	2	20
OL459847	*Taenia* sp. from the present study (TSVN7)	2	2	20
OL459848	*Taenia* sp. from the present study (TSVN8)	2	2	20
OL459849	*Taenia* sp. from the present study (TSVN9)	2	2	20
OL459850	*Taenia* sp. from the present study (TSVN10)	2	2	20
OL459851	*Taenia* sp. from the present study (TSVN11)	2	2	20
OL459852	*Taenia* sp. from the present study (TSVN12)	2	2	20
OL459853	*Taenia* sp. from the present study (TSVN13)	2	2	20
OL459854	*Taenia* sp. from the present study (TSVN14)	2	2	20
OL459855	*Taenia* sp. from the present study (TSVN15)	2	2	20
OL459856	*Taenia* sp. from the present study (TSVN16)	2	2	20
OL459857	*Taenia* sp. from the present study (TSVN17)	2	2	20
OL459858	*Taenia* sp. from the present study (TSVN18)	2	2	20
OL459859	*Taenia* sp. from the present study (TSVN19)	2	2	20
OL459860	*Taenia* sp. from the present study (TSVN20)	2	2	20
OL459861	*Taenia* sp. from the present study (TSVN21)	2	2	20
OL459862	*Taenia* sp. from the present study (TSVN22)	2	2	20
OL459863	*Taenia* sp. from the present study (TSVN23)	2	2	20
OL459864	*Taenia* sp. from the present study (TSVN24)	2	2	20
OL459865	*Taenia* sp. from the present study (TSVN25)	2	2	20
OL459866	*Taenia* sp. from the present study (TSVN26)	2	2	20
OL459867	*Taenia* sp. from the present study (TSVN27)	2	2	20
OL459868	*Taenia* sp. from the present study (TSVN28)	2	2	20
OL459869	*Taenia* sp. from the present study (TSVN29)	2	2	20
OL459870	*Taenia* sp. from the present study (TSVN30)	2	2	20
OL459871	*Taenia* sp. from the present study (TSVN31)	2	2	20
OL459872	*Taenia* sp. from the present study (TSVN32)	2	2	20
OL459873	*Taenia* sp. from the present study (TSVN33)	2	2	20
OL459874	*Taenia* sp. from the present study (TSVN34)	2	2	20
OL459875	*Taenia* sp. from the present study (TSVN36)	2	2	20
OL459876	*Taenia* sp. from the present study (TSVN37)	2	2	20
OL459877	*Taenia* sp. from the present study (TSVN38)	2	2	20
OL459878	*Taenia* sp. from the present study (TSVN39)	2	2	20
TOTAL		26	25	27
