# Supplementary material for: Molecular Identification and Appraisal of the Genetic Variation of Taenia saginata in Central Regions of Vietnam
Source: Life (Basel). 2022 Jan 4;12(1):70. doi: 10.3390/life12010070 (PMC8781798; doi:10.3390/life12010070)

Table S1. Sequences from GenBank. The table reports data on the whole world *Taenia saginata* COI dataset of sequences which were used in the present study and are available on GenBank.

Sample ID	Sampling site	Species	Host	GB Accession #
TSA_BE009	Belgium	*Taenia saginata*	*NOD/Shi-scid mice*	AB107242
TSA_BE164	Belgium	*Taenia saginata*	*Homo sapiens*	AY195858
TSA_BR005	Brazil	*Taenia saginata*	*Homo sapiens*	AB107246
TSA_BR014	Brazil	*Taenia saginata*	*Homo sapiens*	AB107237
TSA_BR137	Brazil	*Taenia saginata*	*Homo sapiens*	AB465238
TSA_CA001	Cambodia	*Taenia saginata*	*Homo sapiens*	AB275143
TSA_CA134	Cambodia	*Taenia saginata*	*Homo sapiens*	AB465241
TSA_CA019	Cambodia	*Taenia saginata*	*Homo sapiens*	MT074050
TSA_CA020	Cambodia	*Taenia saginata*	*Homo sapiens*	MT074049
TSA_CA021	Cambodia	*Taenia saginata*	*Homo sapiens*	MT074048
TSA_CH003	China	*Taenia saginata*	*Homo sapiens*	AB107239
TSA_CH004	China	*Taenia saginata*	*Homo sapiens*	AB107247
TSA_CH015	China	*Taenia saginata*	*Homo sapiens*	AB066495
TSA_CH153	China	*Taenia saginata*	*Homo sapiens*	AB984351
TSA_CH154	China	*Taenia saginata*	*Homo sapiens*	AB984350
TSA_CH155	China	*Taenia saginata*	*Homo sapiens*	AB984349
TSA_CH156	China	*Taenia saginata*	*Homo sapiens*	AB984348
TSA_CH157	China	*Taenia saginata*	*Homo sapiens*	AB984347
TSA_CH158	China	*Taenia saginata*	*Homo sapiens*	AB984346
TSA_CH151	China	*Taenia saginata*	*Homo sapiens*	GU097652
TSA_CH189	China	*Taenia saginata*	*Homo sapiens*	MN452862
TSA_CH002	China	*Taenia saginata*	*Homo sapiens*	MW578279
TSA_EC013	Ecuador	*Taenia saginata*	*Homo sapiens*	AB107238
TSA_EC132	Ecuador	*Taenia saginata*	*Homo sapiens*	AB465243
TSA_ET010	Ethiopia	*Taenia saginata*	*Homo sapiens*	AB107241
TSA_ET130	Ethiopia	*Taenia saginata*	*Homo sapiens*	AB465245
TSA_ET138	Ethiopia	*Taenia saginata*	*Homo sapiens*	AB465237
TSA_ET159	Ethiopia	*Taenia saginata*	*Bos taurus*	AB713906
TSA_ET160	Ethiopia	*Taenia saginata*	*Bos taurus*	AB713905
TSA_ET161	Ethiopia	*Taenia saginata*	*Bos taurus*	AB713904
TSA_FR174	France	*Taenia saginata*	*Homo sapiens*	DQ768207
TSA_IN011	Indonesia	*Taenia saginata*	*Homo sapiens*	AB107240
TSA_IN135	Indonesia	*Taenia saginata*	*Homo sapiens*	AB465240
TSA_IR017	Iran	*Taenia saginata*	*Homo sapiens*	MT998286
TSA_JA047	Japan	*Taenia saginata*	*Homo sapiens*	AB494480
TSA_JA131	Japan	*Taenia saginata*	*Homo sapiens*	AB465244
TSA_JA126	Japan	*Taenia saginata*	*Homo sapiens*	AB645845
TSA_JA125	Japan	*Taenia saginata*	*Bos taurus*	AB644391
TSA_JA050	Japan	*Taenia saginata*	*Homo sapiens*	AB820291
TSA_JA124	Japan	*Taenia saginata*	*Homo sapiens*	AB821273
TSA_KE162	Kenya	*Taenia saginata*	*Homo sapiens*	AM503327
TSA_KE163	Kenya	*Taenia saginata*	*Homo sapiens*	AM503326
TSA_LA024	Laos	*Taenia saginata*	*Homo sapiens*	KY290373
TSA_LA025	Laos	*Taenia saginata*	*Homo sapiens*	KY290372
TSA_LA026	Laos	*Taenia saginata*	*Homo sapiens*	KY290371
TSA_LA027	Laos	*Taenia saginata*	*Homo sapiens*	KY290370

TSA_LA028	Laos	*Taenia saginata*	*Homo sapiens*	KY290369
TSA_LA029	Laos	*Taenia saginata*	*Homo sapiens*	KY290368
TSA_LA030	Laos	*Taenia saginata*	*Homo sapiens*	KY290367
TSA_LA031	Laos	*Taenia saginata*	*Homo sapiens*	KY290366
TSA_LA032	Laos	*Taenia saginata*	*Homo sapiens*	KY290365
TSA_LA033	Laos	*Taenia saginata*	*Homo sapiens*	KY290364
TSA_LA034	Laos	*Taenia saginata*	*Homo sapiens*	KY290363
TSA_LA035	Laos	*Taenia saginata*	*Homo sapiens*	KY290362
TSA_LA036	Laos	*Taenia saginata*	*Homo sapiens*	KY290361
TSA_LA037	Laos	*Taenia saginata*	*Homo sapiens*	KY290360
TSA_LA038	Laos	*Taenia saginata*	*Homo sapiens*	KY290359
TSA_MO003	Mongolia	*Taenia saginata*	*Homo sapiens*	AB271695
TSA_NE008	Nepal	*Taenia saginata*	*Homo sapiens*	AB107243
TSA_PO173	Poland	*Taenia saginata*	*Homo sapiens*	JX402911
TSA_RU049	Russia	*Taenia saginata*	*Homo sapiens*	LC063349
TSA_KO129	South Korea	*Taenia saginata*	*Homo sapiens*	AB465246
TSA_KO016	South-Korea	*Taenia saginata*	*Homo sapiens*	MW750280
TSA_KO022	South Korea	*Taenia saginata*	*Homo sapiens*	MK644931
TSA_KO145	South Korea	*Taenia saginata*	*Homo sapiens*	MK644934
TSA_KO146	South Korea	*Taenia saginata*	*Homo sapiens*	MK644932
TSA_KO147	South Korea	*Taenia saginata*	*Homo sapiens*	MK644933
TSA_KO148	South Korea	*Taenia saginata*	*Homo sapiens*	MK644930
TSA_TH006	Thailand	*Taenia saginata*	*Homo sapiens*	AB107245
TSA_TH007	Thailand	*Taenia saginata*	*Homo sapiens*	AB107244
TSA_TH127	Thailand	*Taenia saginata*	*Homo sapiens*	AB465248
TSA_TH128	Thailand	*Taenia saginata*	*Homo sapiens*	AB465247
TSA_TH133	Thailand	*Taenia saginata*	*Homo sapiens*	AB465242
TSA_TH136	Thailand	*Taenia saginata*	*Homo sapiens*	AB465239
TSA_TH139	Thailand	*Taenia saginata*	*Homo sapiens*	AB465236
TSA_TH140	Thailand	*Taenia saginata*	*Homo sapiens*	AB465235
TSA_TH141	Thailand	*Taenia saginata*	*Homo sapiens*	AB465234
TSA_TH142	Thailand	*Taenia saginata*	*Homo sapiens*	AB465233
TSA_TH143	Thailand	*Taenia saginata*	*Homo sapiens*	AB465232
TSA_TH144	Thailand	*Taenia saginata*	*Homo sapiens*	AB465231
TSA_TH149	Thailand	*Taenia saginata*	*Homo sapiens*	HQ606076
TSA_TH150	Thailand	*Taenia saginata*	*Homo sapiens*	HQ606075
TSA_TH051	Thailand	*Taenia saginata*	*Homo sapiens*	JN986718
TSA_TH052	Thailand	*Taenia saginata*	*Homo sapiens*	JN986717
TSA_TH053	Thailand	*Taenia saginata*	*Homo sapiens*	JN986716
TSA_TH054	Thailand	*Taenia saginata*	*Homo sapiens*	JN986715
TSA_TH055	Thailand	*Taenia saginata*	*Homo sapiens*	JN986714
TSA_TH056	Thailand	*Taenia saginata*	*Homo sapiens*	JN986713
TSA_TH057	Thailand	*Taenia saginata*	*Homo sapiens*	JN986712
TSA_TH058	Thailand	*Taenia saginata*	*Homo sapiens*	JN986711
TSA_TH059	Thailand	*Taenia saginata*	*Homo sapiens*	JN986710
TSA_TH060	Thailand	*Taenia saginata*	*Homo sapiens*	JN986709
TSA_TH061	Thailand	*Taenia saginata*	*Homo sapiens*	JN986708
TSA_TH062	Thailand	*Taenia saginata*	*Homo sapiens*	JN986707
TSA_TH063	Thailand	*Taenia saginata*	*Homo sapiens*	JN986706
TSA_TH064	Thailand	*Taenia saginata*	*Homo sapiens*	JN986705

TSA_TH065	Thailand	*Taenia saginata*	*Homo sapiens*	JN986704
TSA_TH066	Thailand	*Taenia saginata*	*Homo sapiens*	JN986703
TSA_TH067	Thailand	*Taenia saginata*	*Homo sapiens*	JN986702
TSA_TH068	Thailand	*Taenia saginata*	*Homo sapiens*	JN986701
TSA_TH069	Thailand	*Taenia saginata*	*Homo sapiens*	JN986700
TSA_TH070	Thailand	*Taenia saginata*	*Homo sapiens*	JN986699
TSA_TH071	Thailand	*Taenia saginata*	*Homo sapiens*	JN986698
TSA_TH072	Thailand	*Taenia saginata*	*Homo sapiens*	JN986697
TSA_TH073	Thailand	*Taenia saginata*	*Homo sapiens*	JN986696
TSA_TH074	Thailand	*Taenia saginata*	*Homo sapiens*	JN986695
TSA_TH075	Thailand	*Taenia saginata*	*Homo sapiens*	JN986694
TSA_TH076	Thailand	*Taenia saginata*	*Homo sapiens*	JN986693
TSA_TH077	Thailand	*Taenia saginata*	*Homo sapiens*	JN986692
TSA_TH078	Thailand	*Taenia saginata*	*Homo sapiens*	JN986691
TSA_TH079	Thailand	*Taenia saginata*	*Homo sapiens*	JN986690
TSA_TH080	Thailand	*Taenia saginata*	*Homo sapiens*	JN986689
TSA_TH081	Thailand	*Taenia saginata*	*Homo sapiens*	JN986688
TSA_TH082	Thailand	*Taenia saginata*	*Homo sapiens*	JN986687
TSA_TH083	Thailand	*Taenia saginata*	*Homo sapiens*	JN986686
TSA_TH084	Thailand	*Taenia saginata*	*Homo sapiens*	JN986685
TSA_TH085	Thailand	*Taenia saginata*	*Homo sapiens*	JN986684
TSA_TH086	Thailand	*Taenia saginata*	*Homo sapiens*	JN986683
TSA_TH087	Thailand	*Taenia saginata*	*Homo sapiens*	JN986682
TSA_TH088	Thailand	*Taenia saginata*	*Homo sapiens*	JN986681
TSA_TH089	Thailand	*Taenia saginata*	*Homo sapiens*	JN986680
TSA_TH090	Thailand	*Taenia saginata*	*Homo sapiens*	JN986679
TSA_TH091	Thailand	*Taenia saginata*	*Homo sapiens*	JN986678
TSA_TH092	Thailand	*Taenia saginata*	*Homo sapiens*	JN986677
TSA_TH093	Thailand	*Taenia saginata*	*Homo sapiens*	JN986676
TSA_TH094	Thailand	*Taenia saginata*	*Homo sapiens*	JN986675
TSA_TH095	Thailand	*Taenia saginata*	*Homo sapiens*	JN986674
TSA_TH096	Thailand	*Taenia saginata*	*Homo sapiens*	JN986673
TSA_TH097	Thailand	*Taenia saginata*	*Homo sapiens*	JN986672
TSA_TH098	Thailand	*Taenia saginata*	*Homo sapiens*	JN986671
TSA_TH099	Thailand	*Taenia saginata*	*Homo sapiens*	JN986670
TSA_TH100	Thailand	*Taenia saginata*	*Homo sapiens*	JN986669
TSA_TH101	Thailand	*Taenia saginata*	*Homo sapiens*	JN986668
TSA_TH102	Thailand	*Taenia saginata*	*Homo sapiens*	JN986667
TSA_TH103	Thailand	*Taenia saginata*	*Homo sapiens*	JN986666
TSA_TH104	Thailand	*Taenia saginata*	*Homo sapiens*	JN986665
TSA_TH105	Thailand	*Taenia saginata*	*Homo sapiens*	JN986664
TSA_TH106	Thailand	*Taenia saginata*	*Homo sapiens*	JN986663
TSA_TH107	Thailand	*Taenia saginata*	*Homo sapiens*	JN986662
TSA_TH108	Thailand	*Taenia saginata*	*Homo sapiens*	JN986661
TSA_TH109	Thailand	*Taenia saginata*	*Homo sapiens*	JN986660
TSA_TH110	Thailand	*Taenia saginata*	*Homo sapiens*	JN986659
TSA_TH111	Thailand	*Taenia saginata*	*Homo sapiens*	JN986658
TSA_TH112	Thailand	*Taenia saginata*	*Homo sapiens*	JN986657
TSA_TH113	Thailand	*Taenia saginata*	*Homo sapiens*	JN986656
TSA_TH114	Thailand	*Taenia saginata*	*Homo sapiens*	JN986655

TSA_TH115	Thailand	*Taenia saginata*	*Homo sapiens*	JN986654
TSA_TH116	Thailand	*Taenia saginata*	*Homo sapiens*	JN986653
TSA_TH117	Thailand	*Taenia saginata*	*Homo sapiens*	JN986652
TSA_TH118	Thailand	*Taenia saginata*	*Homo sapiens*	JN986651
TSA_TH119	Thailand	*Taenia saginata*	*Homo sapiens*	JN986650
TSA_TH120	Thailand	*Taenia saginata*	*Homo sapiens*	JN986649
TSA_TH121	Thailand	*Taenia saginata*	*Homo sapiens*	JN986648
TSA_TH122	Thailand	*Taenia saginata*	*Homo sapiens*	JN986647
TSA_TH123	Thailand	*Taenia saginata*	*Homo sapiens*	JN986646
TSA_TH048	Thailand	*Taenia saginata*	*Homo sapiens*	HQ606077
TSA_TH039	Thailand	*Taenia saginata*	*Homo sapiens*	KY290358
TSA_TH040	Thailand	*Taenia saginata*	*Homo sapiens*	KY290357
TSA_TH041	Thailand	*Taenia saginata*	*Homo sapiens*	KY290356
TSA_TH042	Thailand	*Taenia saginata*	*Homo sapiens*	KY290355
TSA_TH043	Thailand	*Taenia saginata*	*Homo sapiens*	KY290354
TSA_TH044	Thailand	*Taenia saginata*	*Homo sapiens*	KY290353
TSA_TH045	Thailand	*Taenia saginata*	*Homo sapiens*	KY290352
TSA_TH046	Thailand	*Taenia saginata*	*Homo sapiens*	KY290351

Table S2. Sequences used for PCoA. The table reports data on Vietnamese *Taenia saginata* COI sequences (from the present study) which were used for PCoA, they are categorized according to the host age. We set three different age groups: 0-30 years identified by the code 30, 30-50 years identified by the code 50 and, ≥ 50 years identified by the code 50+.

Group 1		
**Sample Code**	**Country**	**Group of age**
TSVN16	Vietnam	50+
**Group 2**		
**Sample Code**	**Country**	**Group of age**
TSVN4	Vietnam	50+
TSVN5	Vietnam	50+
TSVN8	Vietnam	50+
TSVN9	Vietnam	50+
TSVN11	Vietnam	50+
TSVN12	Vietnam	50+
TSVN15	Vietnam	50+
TSVN17	Vietnam	50+
TSVN18	Vietnam	50+
TSVN19	Vietnam	50+
TSVN20	Vietnam	50+
TSVN21	Vietnam	50+
TSVN24	Vietnam	50+
TSVN27	Vietnam	50+
TSVN30	Vietnam	50+
TSVN32	Vietnam	50+
TSVN36	Vietnam	50+
TSVN39	Vietnam	50+
TSVN13	Vietnam	50
TSVN23	Vietnam	50
TSVN33	Vietnam	50
TSVN34	Vietnam	50
TSVN2	Vietnam	50
TSVN6	Vietnam	50
TSVN7	Vietnam	50
TSVN38	Vietnam	50
TSVN1	Vietnam	30
TSVN3	Vietnam	30
TSVN10	Vietnam	30
TSVN31	Vietnam	30
TSVN37	Vietnam	30
**Group 3**		
**Sample Code**	**Country**	**Group of age**
TSVN25	Vietnam	50+
TSVN28	Vietnam	50+
TSVN29	Vietnam	50+
TSVN14	Vietnam	50
TSVN26	Vietnam	50
TSVN22	Vietnam	30
TSVN25	Vietnam	50+

Table S3. Sequences for PCoA. The table reports data on the *Taenia saginata* COI sequences which were used for PCoA in the present study and are available on GenBank.

Group 1		
**GenBank #**	**Country**	**Collection year**
AB275143	Cambodia	2007
MW578279	China	2021
AB271695	Mongolia	2007
AB107247	China	2004
AB107246	Brazil	2004
AB107245	Thailand	2004
AB107244	Thailand	2004
AB107243	Nepal	2004
AB107242	Belgium	2004
AB107241	Ethiopia	2004
AB107240	Indonesia	2004
AB107239	China	2004
AB107238	Ecuador	2004
AB107237	Brazil	2004
AB066495	China	2004
MW750280	South-Korea	2002
MT998286	Iran	2020
MT074050	Cambodia	2020
MT074049	Cambodia	2020
MT074048	Cambodia	2020
MK644931	South-Korea	2018
KY290373	Laos	2017
KY290372	Laos	2017
KY290371	Laos	2017
KY290370	Laos	2017
KY290369	Laos	2017
KY290368	Laos	2017
KY290367	Laos	2017
KY290366	Laos	2017
KY290365	Laos	2017
KY290364	Laos	2017
KY290363	Laos	2017
KY290362	Laos	2017
KY290361	Laos	2017
KY290360	Laos	2017
KY290359	Laos	2017
KY290358	Thailand	2017
KY290357	Thailand	2017
KY290356	Thailand	2017
KY290355	Thailand	2017
KY290354	Thailand	2017
KY290353	Thailand	2017
KY290352	Thailand	2017
KY290351	Thailand	2017
AB494480	Japan	2009

LC063349	Russia	2016
AB820291	Japan	2012
JN986718	Thailand	2013
JN986717	Thailand	2013
JN986716	Thailand	2013
JN986715	Thailand	2013
JN986714	Thailand	2013
JN986713	Thailand	2013
JN986712	Thailand	2013
JN986711	Thailand	2013
JN986710	Thailand	2013
JN986709	Thailand	2013
JN986708	Thailand	2013
JN986707	Thailand	2013
JN986706	Thailand	2013
JN986705	Thailand	2013
JN986704	Thailand	2013
JN986703	Thailand	2013
JN986702	Thailand	2013
JN986701	Thailand	2013
JN986700	Thailand	2013
JN986699	Thailand	2013
JN986698	Thailand	2013
JN986697	Thailand	2013
JN986696	Thailand	2013
JN986695	Thailand	2007
JN986694	Thailand	2021
JN986693	Thailand	2007
JN986692	Thailand	2004
JN986691	Thailand	2004
JN986690	Thailand	2004
JN986689	Thailand	2004
JN986688	Thailand	2004
JN986687	Thailand	2004
JN986686	Thailand	2004
JN986685	Thailand	2004
JN986684	Thailand	2004
JN986683	Thailand	2004
JN986682	Thailand	2004
JN986681	Thailand	2004
JN986680	Thailand	2002
JN986679	Thailand	2020
JN986678	Thailand	2020
JN986677	Thailand	2020
JN986676	Thailand	2020
JN986675	Thailand	2018
JN986674	Thailand	2017
JN986673	Thailand	2017
JN986672	Thailand	2017

JN986671	Thailand	2017
JN986670	Thailand	2017
JN986669	Thailand	2017
JN986668	Thailand	2017
JN986667	Thailand	2017
JN986666	Thailand	2017
JN986665	Thailand	2017
JN986664	Thailand	2017
JN986663	Thailand	2017
JN986662	Thailand	2017
JN986661	Thailand	2017
JN986660	Thailand	2017
JN986659	Thailand	2017
JN986658	Thailand	2017
JN986657	Thailand	2017
JN986656	Thailand	2017
JN986655	Thailand	2017
JN986654	Thailand	2017
JN986653	Thailand	2017
JN986652	Thailand	2017
JN986651	Thailand	2009
JN986650	Thailand	2016
JN986649	Thailand	2012
JN986648	Thailand	2013
JN986647	Thailand	2013
JN986646	Thailand	2013
AB821273	Japan	2013
AB644391	Japan	2013
AB645845	Japan	2013
AB465248	Thailand	2013
AB465247	Thailand	2013
AB465246	South-Korea	2013
AB465245	Ethiopia	2013
AB465244	Japan	2013
AB465243	Ecuador	2013
AB465242	Thailand	2013
AB465241	Cambodia	2013
AB465240	Indonesia	2013
AB465239	Thailand	2013
AB465238	Brazil	2013
AB465237	Ethiopia	2013
AB465236	Thailand	2013
AB465235	Thailand	2013
AB465234	Thailand	2013
AB465233	Thailand	2013
AB465232	Thailand	2013
AB465231	Thailand	2010
MK644934	South-Korea	2019
MK644932	South-Korea	2019

MK644933	South-Korea	2019
MK644930	South-Korea	2019
GU097652	China	2016
AB984351	China	2014
AB984350	China	2014
AB984349	China	2014
AB984348	China	2014
AB984347	China	2014
AB984346	China	2014
AB713906	Ethiopia	2012
AB713905	Ethiopia	2012
AB713904	Ethiopia	2012
AM503327	Kenya	2007
AM503326	Kenya	2007
AY195858	Belgium	2006
JX402911	Poland	2012
DQ768207	France	2016
MN452862	China	2019
TSVN1	Vietnam	2019
TSVN2	Vietnam	2019
TSVN3	Vietnam	2019
TSVN4	Vietnam	2019
TSVN5	Vietnam	2019
TSVN6	Vietnam	2019
TSVN7	Vietnam	2019
TSVN8	Vietnam	2019
TSVN9	Vietnam	2019
TSVN10	Vietnam	2019
TSVN11	Vietnam	2019
TSVN12	Vietnam	2019
TSVN13	Vietnam	2019
TSVN14	Vietnam	2019
TSVN15	Vietnam	2019
TSVN16	Vietnam	2019
TSVN17	Vietnam	2019
TSVN18	Vietnam	2019
TSVN19	Vietnam	2019
TSVN20	Vietnam	2019
TSVN21	Vietnam	2019
TSVN22	Vietnam	2019
TSVN23	Vietnam	2019
TSVN24	Vietnam	2020
TSVN25	Vietnam	2020
TSVN26	Vietnam	2020
TSVN27	Vietnam	2020
TSVN28	Vietnam	2020
TSVN29	Vietnam	2020
TSVN30	Vietnam	2020
TSVN31	Vietnam	2020

TSVN32	Vietnam	2020
TSVN33	Vietnam	2020
TSVN34	Vietnam	2020
TSVN36	Vietnam	2020
TSVN37	Vietnam	2020
TSVN38	Vietnam	2020
TSVN39	Vietnam	2020
**Group 2**		
**GenBank #**	**Country**	**Collection year**
HQ606075	Thailand	2010
HQ606076	Thailand	2010
HQ606077	Thailand	2016

Figure S1. Principal Coordinates Analysis (PCoA) performed on the Vietnamese *Taenia saginata* COI dataset. Bi-dimensional plot shows the genetic differentiation among specimens due to the nucleotide substitutions per site found in the dataset. The number 1 inside the figure stands for Group 1, the number 2 stands for Group 2, and the number 3 stands for Group 3 (see Table S2 for details). PCoA1 (axis X) accounts for 41.99% of variation, while PCoA2 (axis Y) accounts for 10.97% of variation.

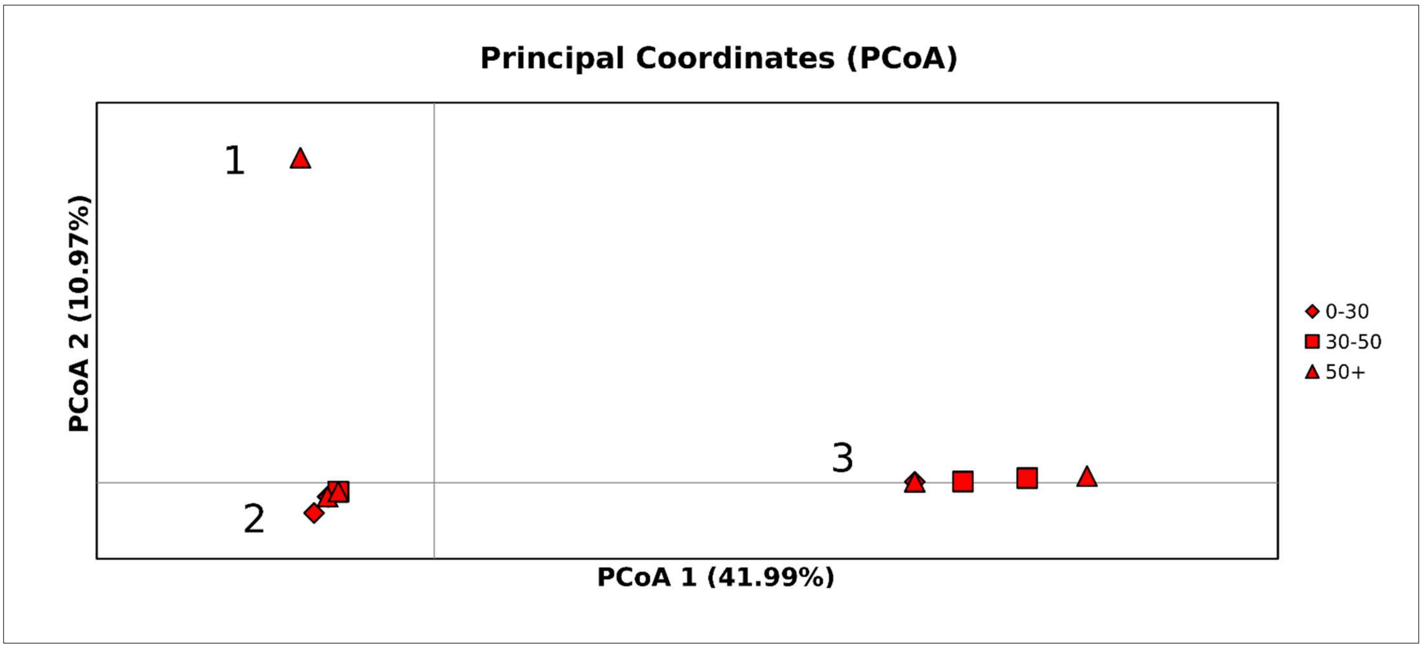

Supplement: Supplementary file 1 [file life-12-00070-s001.zip › life-1494908-supplementary.pdf]
